# Efficacy of fulvestrant 500 mg in Chinese postmenopausal women with advanced/recurrent breast cancer and factors associated with prolonged time-to-treatment failure: A retrospective case series

**DOI:** 10.1097/MD.0000000000020821

**Published:** 2020-07-17

**Authors:** Jian Huang, Ping Huang, Xi-ying Shao, Yan Sun, Lei Lei, Cai-jin Lou, Wei-wu Ye, Jun-qing Chen, Wen-ming Cao, Yuan Huang, Ya-bing Zheng, Xiao-jia Wang, Zhan-hong Chen

**Affiliations:** Breast Medical Oncology, Cancer Hospital of the University of Chinese Academy of Sciences (Zhejiang Cancer Hospital), Hangzhou, P.R. China.

**Keywords:** advanced breast cancer, endocrine therapy, fulvestrant, metastases, postmenopausal, time-to-treatment failure

## Abstract

This study was to investigate the efficacy and safety of fulvestrant 500 mg for the treatment of hormone receptor positive advanced postmenopausal women, including ovarian ablation and investigated factors associated with prolonged time-to-treatment failure.

Data from 60 women with metastatic breast cancer who were treated at Zhejiang Cancer Hospital. Patients received 500 mg (n = 60) between December 2011 and November 2012 were followed until November 2017. Main outcomes were clinical responses to fulvestrant, including best response, progressive disease, partial response, and stable disease lasting 12 months or more. Time to progression and time to progression-free-survival were also analyzed.

Among the included 60 patients (mean age 47.18 years), 51 (85.0%) had received prior adjuvant therapy. During follow-up after fulvestrant treatment, the median PFS for the best response was derived as 7.0 months (inter-quartile = 4, 13.8 months). The observed median progression-free-survival time for best response was represented longer when fulvestrant was first-line treatment than when patients received prior endocrine and/or chemotherapy. Univariate analysis revealed that receiving either endocrine therapy only or endocrine therapy plus chemotherapy prior to fulvestrant treatment may be associated with median progression-free survival time to best response (*P* = .002, .026, .007, respectively).

Fulvestrant treatment is safe and well-tolerated in women with hormone-sensitive advanced breast cancer, and first-line fulvestrant therapy increases progression-free-survival time, especially in patients without prior adjuvant treatment.

## 1 Introduction

Breast cancer is the most frequently diagnosed cancer in women, affecting 1.38 million women worldwide, and is a leading cause of cancer deaths in women, due mainly to metastases.^[1]^ Patients with metastatic breast cancer, some presenting initially with metastases and about 30% developing distant metastases later, have a median survival of only 2 to 3 years.^[2]^ Although breast cancer deaths have decreased as a result of increased screening begun in the 1990s, Stage IV metastatic disease, regardless of when it occurs in the disease course, is almost always incurable.^[1,2]^

Since estrogen is responsible for the normal physiology of the female sex organs, hormone sensitivity is the primary predictor of patients’ response to therapy when treating breast neoplasms. Breast cancer progresses when over-expressed estrogen receptors (ER) increase transcriptional activity.^[3]^ Treatment of advanced metastatic disease, therefore, will try to reduce circulating estrogens using aromatase inhibitors or block the receptors using selective ER modulators. The effects of these endocrine therapies, however, are limited in many patients (about 50%) with metastatic disease who develop acquired resistance, leading in turn to endocrine insensitivity and the increased migration of differentiated epithelial cancer cells.^[3]^ ER status is the main predictor of patients’ response to endocrine therapy.^[4]^

Treatment options for breast cancer are determined by tumor staging at diagnosis and predicted the risk of recurrence.^[1]^ Sequential endocrine treatment is still considered the preferred strategy for treating hormone-sensitive metastatic breast cancer, and new treatment regimens are prescribed when progression occurs.^[5]^ Endocrine treatment may include selective estrogen receptor modulators (SERMS) such as tamoxifen, toremifene and fulvestrant, and aromatase inhibitors such as anastrozole.^[5,6]^ Other drugs, including the CDK4/6 inhibitors palbociclib and ribociclib, and pictilisib and buparlisib that target the phosphatidylinositol-3-kinase (PI3K)/Akt and the mammalian target of rapamycin (mTOR) signaling (PI3K/AKt/mTOR) pathway, are sometimes used in conjunction with fulvestrant. Such combination endocrine therapy has been suggested to be more effective for hormone-sensitive breast cancer than monotherapy because it balances the benefits of endocrine therapy while also managing toxicity levels.^[7]^

Fulvestrant 500 mg is a selective ER antagonist that downregulates cellular ER,^[8]^ resulting in complete inhibition of estrogen signaling through the ER [Nathan]. It is Food and Drug Administration (FDA)-approved for treating postmenopausal women with hormone receptor-positive metastatic breast cancer after failure of prior endocrine therapies. It has been shown to be effective and well tolerated in the defined population, regardless of the number of prior endocrine therapies.^[9]^ A recent review of trials evaluating fulvestrant treatment for breast cancer reported comparable efficacy between fulvestrant 250 mg every 28 days and anastrozole, tamoxifen, and exemestane; however, PFS was improved compared with anastrozole when fulvestrant 500 mg was given with an extra loading dose.^[7]^ Results of multiple studies show fulvestrant to be an important endocrine therapy, given either alone or combined with other agents. Trials continue to evaluate fulvestrant 500 mg for treating metastatic breast cancer in postmenopausal women. However, selecting the most appropriate endocrine treatment to manage metastatic breast cancer and improve survival remains a challenge for prescribing physicians.^[6,10]^

The present study aimed to report the authors’ experience with fulvestrant 500 mg in treating postmenopausal women with advanced or recurrent breast cancer that had progressed after prior endocrine therapies. We also sought to investigate factors associated with prolonged time-to-treatment failure in this population.

## 2 Methods

### 2.1 Study design and sample

This retrospective study was conducted of data from a total of 60 postmenopausal women with metastatic breast cancer who were treated at the Zhejiang Cancer Hospital. All included patients had comprehensive baseline demographic and clinical information, including at least one imaging evaluation prior to diagnosis of metastatic breast cancer. All patients were treated with fulvestrant 500 mg between December 2011 and November 2012. This is a study which retrospectively examined and routinely collected data over a 5-year period to November 2017. By the time we started this study, all of the patients had started or stopped the fulvestrant treatment.

### 2.2 Ethical considerations

After the study purpose and procedure were explained, all 60 patients provided signed informed consent for their data to be evaluated in a later study and reported anonymously. The study protocol was approved by the Institutional Review Board of Zhejiang Cancer Hospital.

### 2.3 Treatment protocol

Most patients (85%) in the study sample had been pretreated with endocrine therapies or chemotherapies prior to receiving fulvestrant therapy. During the treatment period, patients were given high-dose (500 mg, for 5 mL intramuscular injections twice) of fulvestrant for 2 groups, one in each buttock, on days 0, 14, and 28 and every 28 days thereafter, as previously described.^[10]^

Two prescriptions for fulvestrant were shown as follows: one is 250 mg, intramuscular injection for 1 time (i.e., conventional dose); the other is 250 mg, intramuscular injection for 2 times (i.e., high dose). Because the high-dose (500 mg, for injections twice) was found in later studies, it was significantly more effective than the conventional dose (from the Phase II FIRST study and the Phase III FALCON study). Therefore, US FDA approved high-dose indications. In this study, only 2 patients chose the usual dose of treatment, rather than 2 groups of patients. Data from patients receiving fulvestrant 250 mg were not included into the efficacy and safety analysis. This study was a hormone receptor-positive breast cancer endocrine therapy at late stage, not an initial postoperative adjuvant endocrine therapy. Therefore, it was not needed to divide breast cancer into Luminal A and Luminal B subtypes according to the level of Ki67, a cellular marker for proliferation, expression to predict the risk of recurrence. The selection criteria for endocrine therapy for breast cancer at late stage can be applied as long as the hormone receptor expression is detected once in the patient's medical history, and even estrogen receptor (ER) or progesterone receptor (PgR) can be used. Of course, the expression of hormones in patients with metastatic lesions has a certain effect on the efficacy of endocrine therapy.

### 2.4 Outcome measures

Patients’ clinical responses to fulvestrant were evaluated using response evaluation criteria for solid tumors every 3 months according to the RECIST 1.1 criteria. Clinical status of best response was defined as the average of all patients experiencing progressive disease (PD), partial response (PR), or stable disease (SD) lasting ≥12 months. The time to progression (TTP) and time to progression-free-survival (PFS) were also analyzed retrospectively. TTP was defined as the time from the date of initial fulvestrant treatment to the last follow-up date of progression or death. PFS was defined as progression-free survival time of the best response from fulvestrant treatment. AE were measured according to the common terminology criteria for Adverse Events, version 4.0.

### 2.5 Statistical analysis

Baseline characteristics are presented as mean ± standard deviation (STD) and range (min. to max.) for continuous variables and n (%) for categorical variables. AE are presented as number of patients. Patients’ best response was summarized as n/N for progressive disease response status for given clinical status; and the respective progression-free-survival (PFS) time with the best response was summarized as median with inter-quartile (IQR) for given clinical characteristics and compared using either Mann–Whitney *U* test or Kruskall–Wallis test to identify associations. The PFS curve is also presented using Kaplan–Meier curve (the event was set as patients with non-PD, i.e., PR or SD) and compared with line of fulvestrant treatments using the log-rank test.

During patient follow-up, TTP for all patients is presented using the Kaplan–Meier curve. For TTP, events were set for patients with the response of PD and the TTP time was derived from initial treatment with fulvestrant to PD occurred for patients, or to the last follow-up for those without PD. A mean with 95% confidence intervals (CI) was summarized along with the Kaplan–Meier curve. All statistical assessments were two-tailed and considered statistically significant at *P* < .05. All statistical analyses were carried out with IBM SPSS statistical software version 22 for Windows (IBM Corp., Armonk, New York, NY).

## 3 Results

A total of 60 women with metastatic breast cancer and a mean age of 47.18 years (range: 24–73 years) were enrolled in this study. During the study, 60 patients received fulvestrant 500 mg. Mean age at metastasis was 51.76 years (range: 28–79 years). Among the 60 patients, 52 patients (86.67%) were postmenopausal; 13 patients (21%) had ovarian resection surgery before or during fulvestrant treatment; 40 patients (66.67%) were dual hormone-receptor positive (estrogen receptor positive/progesterone receptor positive, or ER+/PR+), 6 (10.00%) were ER+PR-, 4 (6.67%) were ER-PR+, and 10 (16.67%) were ER+ and PR status was unknown; all patients were HER2-negative; 51 patients (85.0%) had received adjuvant therapy (endocrine- and/or chemo-therapy: see details below); all patients had metastasis, including 22 with metastasis of visceral organs and all others with metastasis of non-visceral organs.

Regarding adjuvant therapy, among the 60 patients, 9 patients (15.00%) had received endocrine therapy only, 4 patients (6.70%) had received chemotherapy only, 31 patients (51.70%) received both endocrine and chemotherapy, and the other 16 patients (26.70%) were treatment naïve. Among all patients, 21 patients (35.0%) were treated with fulvestrant as first-line therapy, 24 (40.0%) as second-line, and 15 (25.0%) as the third-line or above treatments. In patients who had received both endocrine and chemotherapy prior to fulvestrant treatment, using fulvestrant as the first-line therapy has 17 patients (28.33%), second-line has 6 patients (10.00%), and third-line or above has 37 patients (61.67%). (Table 1).

**Table 1:**
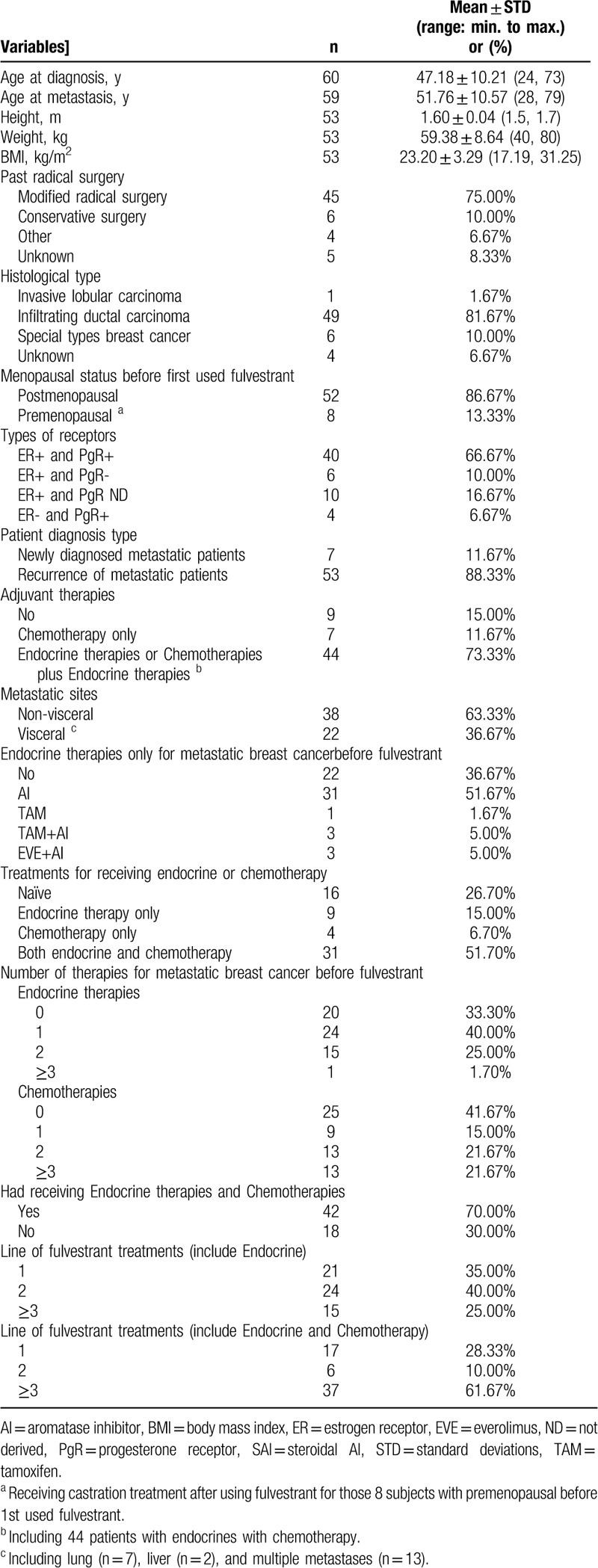
Summary of patients’ demographics and clinical characteristics.

Patients’ best response and AE during follow-up are summarized in Tables 2 and 3, respectively. Three patients died during the follow-up period and their survival times were recorded as 12 months, 16.3 months, and 62 months after receiving treatment. For the best response status, 14 patients (23.3%) had the partial disease (PD), 5 (8.3%) had the partial response (PR), and 41 (68.3%) had the stable disease (SD) (Table 2). AE included 12 patients with at least one treatment-related AE, 5 were possibly related, and 6 were not related. One patient asked for treatment to be discontinued after the AE (Table 3).

**Table 2:**
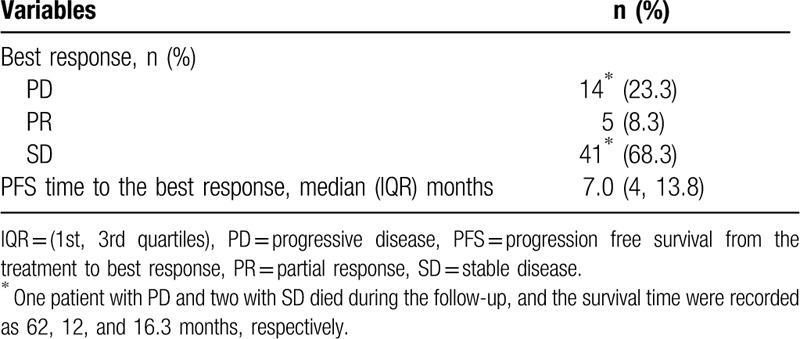
Summary of the status of best response, and PFS time of those 60 patients.

**Table 3:**
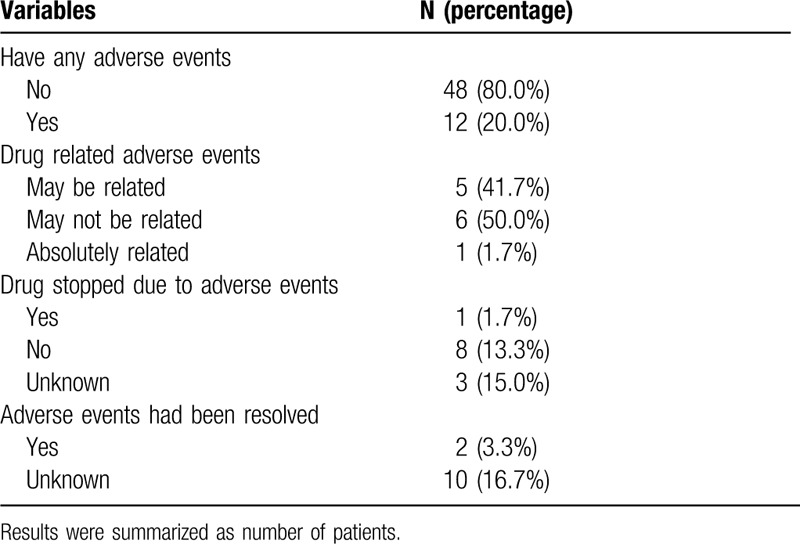
Summary of the adverse events.

**Figure 1:**
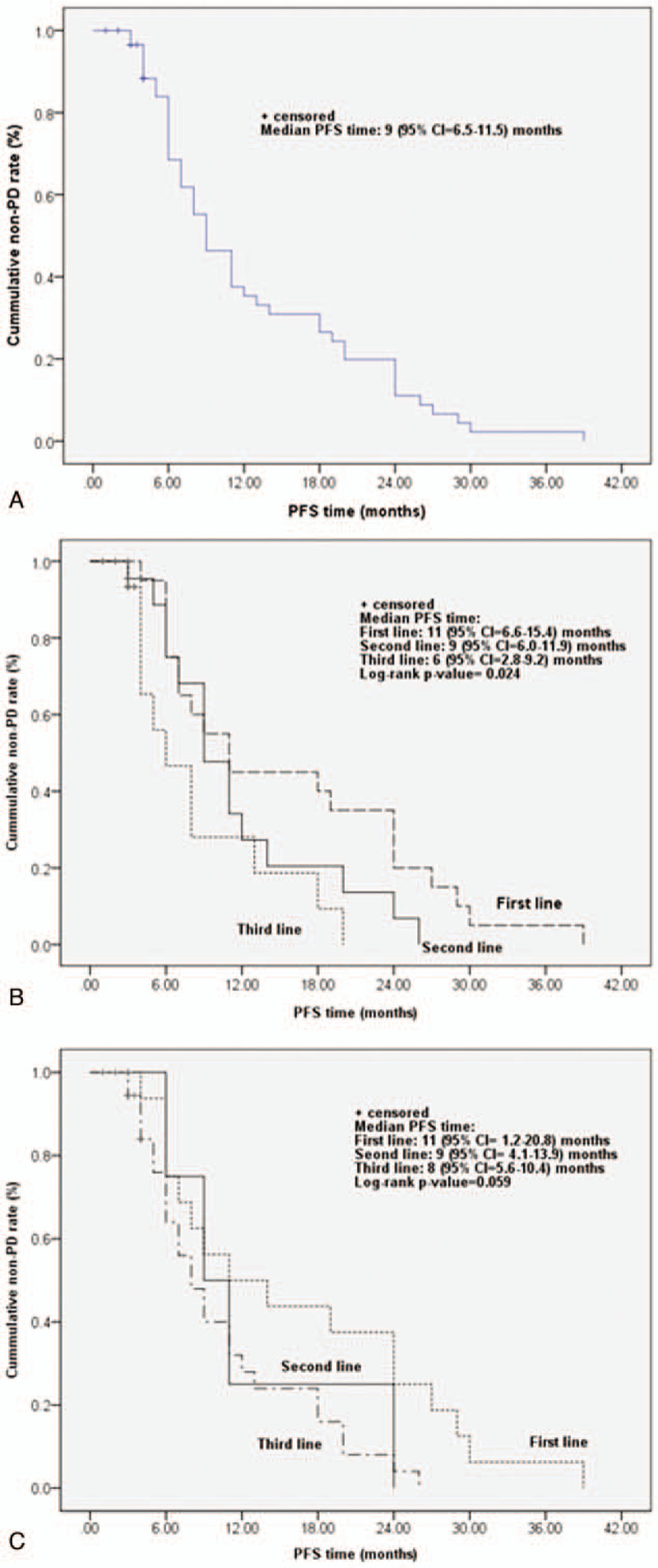
Kaplan–Meier curve of PFS time curve from the treatment to best response for (A) all patients, (B) comparing with line of fulvestrant (include Endocrine), and (C) comparing with line of fulvestrant (include Endocrine + Chemotherapy). Event for Kaplan–Meier curve was set as non-PD (PR+SD) during the follow-up. The PFS time was the time from the treatments to best-response and was presented as estimated median with 95% confidence (95% CI.). Log-rank test was used to compare the difference among line of fulvestrant treatments. PD = progression disease, PFS = progression-free-survival, PR = partial response, SD = stable disease.

**Figure 2:**
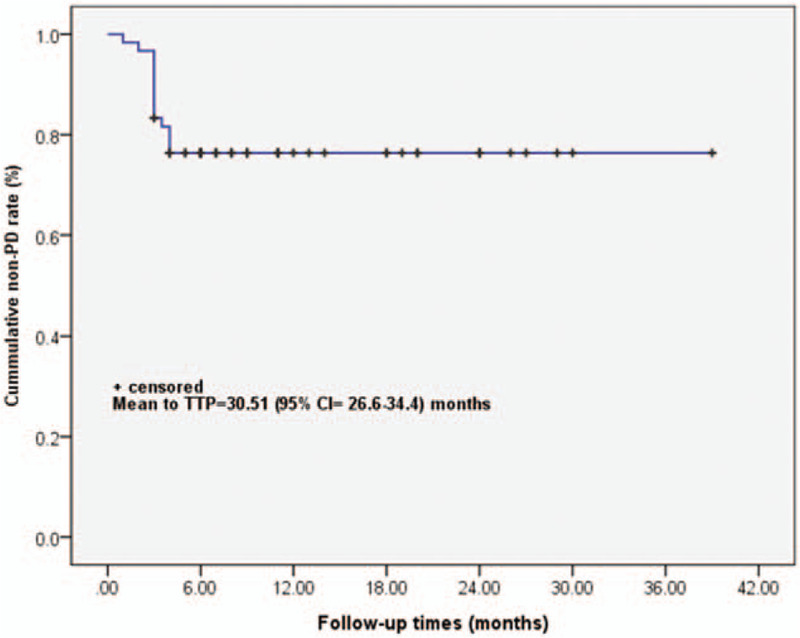
Kaplan–Meier curve of TTP curve in the 60 patients at the last follow-up. Event for Kaplan–Meier curve was set as PD during the follow-up. The TTP was presented as estimated mean with 95% confidence (95% CI). The event for Kaplan–Meier curve was set as patients either with PD or dead occurred during the follow-up. PD = progression disease, TTP = time to progression.

Table 4 shows results of univariate analysis of associations between median PFS time to best response and baseline characteristics of 60 patients. For patients receiving endocrine therapies only for metastatic breast cancer or endocrine therapy plus chemotherapy prior to receiving fulvestrant for metastatic breast cancer, prior endocrine therapy may be associated with median PFS time to best response (*P* = .002, .026, .007, respectively) (Table 4). The Kaplan–Meier curve of PFS time is graphed in Fig. 1 and the estimated median PFS time was 9 months (95%CI = 6.5–11.5 months) (Fig. 1A). The log-rank test shows that the PFS time curve was significantly associated with the line of fulvestrant treatment (including endocrine therapy) (*P* = .024) (Fig. 1B). The median PFS time of those who treated Fulvestrant (include Endocrine) as the first, second, and third, or over third lines were 9, 6, and 4 months, respectively. The median PFS time of those who treated Fulvestrant (include endocrine and chemotherapy) as the first, second, and third, or over third lines were 10, 4.5, and 5.5 months, respectively. The Kaplan–Meier curve of time to progression (TTP) is graphed in Fig. 2 and the estimated mean TTP was 30.51 months (95%CI = 26.6–34.4 months).

**Table 4:**
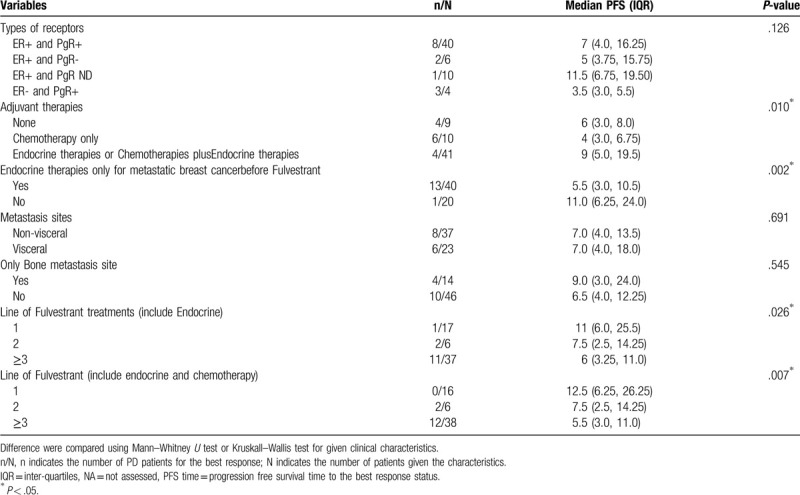
Univariate analysis of association of median PFS time to the best response of the 60 patients with considering the baseline characteristics.

## 4 Discussion

In the present study, during the 5-year follow-up of postmenopausal women with advanced or recurrent breast cancer who were treated with fulvestrant 500 mg as first-line therapy, the median PFS time was 9 months, and the median PFS time were also derived as 6 and 4 months for those treated as second line and ≥3rd line, respectively. Additionally, the PFS times were longer in patients receiving first-line fulvestrant treatment than in those who received sequential second- or third-line endocrine treatment with fulvestrant.

Results of the present study are similar to those of some previous studies and clinical trials of fulvestrant 500 mg. In China, besides the FDA approval of fulvestrant 500 mg for postmenopausal women with metastatic/recurrent breast cancer, a phase III registration trial in China found that fulvestrant 250 mg was effective in the same population.^[11]^ Thereafter, when a study in China investigated the safety and efficacy of fulvestrant 250 mg versus 500 mg, fulvestrant 500 mg was found to have superior clinical benefit in patients with ER-positive, locally advanced or metastatic breast cancer with disease progression after prior endocrine or chemo- therapy.^[12]^ Fulvestrant therapy was also shown to be effective for advanced/recurrent breast cancer in patients with fewer or no prior chemotherapy treatments and no liver involvement.^[13]^ In a phase II trial comparing overall survival (OS) after first-line fulvestrant 500 mg treatment versus anastozole for postmenopausal women with ER-positive breast cancer, Ellis et al^[14]^ initially found that TTP was longer in women receiving fulvestrant 500 mg, suggesting that OS was also longer compared to that with anastozole treatment; this was the first report of fulvestrant monotherapy showing improved efficacy compared with an aromatase inhibitor (AI). However, for confirmation of OS, those authors deferred to the then ongoing phase III FALCON study,^[10]^ which later showed that PFS was significantly longer in the fulvestrant group compared with the group receiving anastozole, and median PFS was 16.6 months in women with advanced or metastatic breast cancer who had not received prior endocrine therapy. Although the present study did not measure OS directly, we did find that TTP and PFS were significantly longer in patients who had not received prior endocrine or other therapies. The median PFS in the present study was 9 months in patients receiving first-line fulvestrant treatment, an important clinical improvement. However, a recent review of the literature^[15]^ reports that few patients with locally advanced or metastatic breast cancer are treatment naïve and additional studies are needed to evaluate first-line fulvestrant treatment in this population.

Results of the present study are also remarkably different from results of certain other studies comparing combination endocrine–endocrine treatment to fulvestrant 500 mg alone. Evidence reported by Johnston et al^[16]^ showed no benefit for either fulvestrant or anastrozole in hormone-receptor-positive breast cancer when compared with first-line fulvestrant treatment alone. Those authors also reported that endocrine treatment had a median PFS of only about 3 to 4 months, indicating limited, if any, efficacy for fulvestrant. In another study of fulvestrant plus anastrozole versus fulvestrant treatment alone, no significant differences were found in TTP between the 2 regimens.^[17]^ These results may possibly be explained by suboptimal dosing of fulvestrant, since many other studies show more favorable outcomes. Combining fulvestrant treatment with CDK4/6 inhibitors palbociclib appeared to be more effective (PFS 9.5 months vs 4.6 months with placebo) even in the presence of endocrine resistance from prior treatments.^[6]^

Regardless of benefits of fulvestrant treatment in patients with metastatic/recurrent breast cancer who did not receive prior endocrine or other therapies, sequential endocrine therapy continues to be recommended by some authors,^[18]^ primarily for premenopausal women for whom combining endocrine therapies was shown to be superior to monotherapy^[19]^ compared with its use in postmenopausal women. Iwase et al^[18]^ favor the use of a subsequent therapy with a different mechanism of action to the prior therapy for extending survival in hormone-sensitive breast cancer. This seems to indicate that patients who are treated successfully with an estrogen therapy are more likely to respond to re-estrogen deprivation therapy, but in the present study and other studies, failure of prior therapies—either endocrine or chemotherapy—did not lead to successful outcomes with fulvestrant 500 mg. However, Nathan et al^[7]^ suggest that fulvestrant could be the “endocrine backbone” of combination therapy because of its ability to overcome gene mutations in relapsed patients or those who received adjuvant aromatase inhibitors. Still other authors support the use of maintenance endocrine therapy to prolong response and delay relapse after prior chemotherapy in women with ER-positive metastatic breast cancer; although this sequence has no advantage in terms of survival, TTP may show modest improvement.^[20]^ The cellular receptor signaling pathways that interact with ER are shown to play a role in the resistance to endocrine therapy in ER-positive metastatic breast cancer.^[21]^

In terms of prolonged time to treatment failure, results of the present study suggest that receiving either endocrine therapy only (fulvestrant first-line treatment) or endocrine therapy plus chemotherapy prior to fulvestrant treatment may be associated with median PFS time to best response, although these results were without statistical significance. Araki et al^[13]^ suggest that the duration of prior endocrine therapy may not be predictive of the TTF of fulvestrant 500 mg treatment when patients have received heavy sequential endocrine therapy and/or chemotherapy. In either case, prior failure of endocrine therapy or other therapies appears to be a factor in the outcomes of postmenopausal women with advanced/recurrent breast cancer who are subsequently treated with fulvestrant, which further complicates treatment decisions.

Dosing may also be an important consideration when deciding how to use fulvestrant most effectively. The dosage of fulvestrant 500 mg as used in the FALCON trial^[10]^ is applied most often and was used in the present study, consisting of 2 n-mL intramuscular injections of fulvestrant 500 mg, one in each buttock, on days 14 and 28 and then monthly thereafter. When a small trial with 144 patients compared dosages of 250 mg per month; a loading dose of 500 mg on day 0 followed by 250 mg doses on days 14 and 28 and monthly thereafter; and a high dose of 500 mg on days 0, 14, and 28 and monthly thereafter, which is closest to the FALCON dosage,^[10]^ no significant differences in overall response rate were found between the 3 groups, although all regimens were well tolerated.^[22]^ Higher doses of fulvestrant, however, are associated with increased down-regulation of ER, reducing ER expression by at least 25%, which downregulates proliferation of cancer cells.^[23]^

### 4.1 Limitations

This study has several limitations, including that it is a retrospective study and causality cannot be inferred from the results. The sample size is relatively small and subjects were recruited from only one cancer center in China, so results may not be generalized to other institutions or locations. Furthermore, the outcomes of patients on sequential hormonal therapy after fulvestrant treatment should be investigated. Additional prospective study is needed to corroborate findings from the present study and to further explore the efficacy of fulvestrant in treating breast cancer patients who have experienced disease progression after previous treatment with endocrine therapy and/or chemotherapy.

## 5 Conclusions

Fulvestrant treatment is safe and well tolerated with demonstrated efficacy in postmenopausal women with hormone-sensitive advanced breast cancer, and PFS time is increased with first-line fulvestrant therapy, especially in patients without prior adjuvant treatment. However, it remains unclear whether combined endocrine treatment offers any additional benefit than first-line fulvestrant treatment alone for patients with acquired resistance from previous endocrine or other therapies. Results suggest that endocrine therapy only or endocrine therapy plus chemotherapy prior to fulvestrant treatment may be associated with median PFS time to best response, although these results were without statistical significance.

## Author contributions

**Conceptualization:** Ping Huang, Xi-ying Shao, Cai-jin Lou, Wen-ming Cao, Yuan Huang, Ya-bing Zheng, Xiao-jia Wang, Zhan-hong Chen.

**Data curation:** Xi-ying Shao, Cai-jin Lou, Wei-wu Ye, Jun-qing Chen, Ya-bing Zheng, Xiao-jia Wang.

**Formal analysis:** Jian Huang, Ping Huang, Xi-ying Shao, Yan Sun, Wei-wu Ye, Jun-qing Chen, Wen-ming Cao, Xiao-jia Wang, Zhan-hong Chen.

**Funding acquisition:** Jun-qing Chen.

**Investigation:** Jian Huang, Wen-ming Cao.

**Methodology:** Jian Huang, Yan Sun, Lei Lei, Yuan Huang.

**Resources:** Lei Lei.

**Supervision:** Jian Huang.

**Visualization:** Yuan Huang.

**Writing – original draft:** Jian Huang, Ya-bing Zheng.

**Writing – review & editing:** Jian Huang, Zhan-hong Chen.
